# The role of cage height on the flexibility and load sharing of lumbar spine after lumbar interbody fusion with unilateral and bilateral instrumentation: a biomechanical study

**DOI:** 10.1186/s12891-017-1845-1

**Published:** 2017-11-21

**Authors:** Lin Du, Xiao-jiang Sun, Tang-jun Zhou, Yuan-chao Li, Chen Chen, Chang-qing Zhao, Kai Zhang, Jie Zhao

**Affiliations:** 1 Shanghai Key Laboratory of Orthopedic Implants, Department of Orthopedics, Shanghai Ninth People’s Hospital, Shanghai Jiao Tong University School of Medicine, 639 Zhizaoju Road, Shanghai, 200011 People’s Republic of China; 2 School of Mechanical Engineering, Shanghai Jiao Tong University, 800 Dongchuan Road, Shanghai, 200240 People’s Republic of China

**Keywords:** Lumbar spine, Stability, Flexibility, Load sharing, Unilateral fixation, Bilateral fixation, Interbody fusion

## Abstract

**Background:**

One- and two-level lumbar interbody fusion with unilateral instrumentation is as effective as that with bilateral instrumentation. The height of the interbody cage influences the operated segment stability and the fusion technique success. The purpose of this research was to determine the effect of the fusion cage height (i.e. long and short) on both the stability (based on flexibility measures) and load sharing of the unilateral and bilateral instrumented transforaminal lumbar interbody fusion (TLIF) technique.

**Methods:**

The flexibility and load sharing tests were performed on seven human lumbar spines. Different configurations combining a long or short cage with a unilateral, bilateral, or no posterior fixation were used to stabilize the operated segment. Two sets of modular cages were designed for each type of test to simulate the long and short cages. During the flexibility test, a pure-moment load of 7.5 Nm was applied. The range of motion (ROM) was recorded for flexion–extension, lateral bending, and axial rotation. During the load sharing test, an axial-compression load of 400 N was applied. The load bearing of the cages was recorded using a cage-embedded load cell.

**Results:**

When the fusion cage height decreased 2 mm, the segment flexibility with unilateral fixation showed a significant increase in the ROM for flexion–extension, lateral bending, and axial rotation of 74.9, 83.8, and 175.2% (*P* < 0.01), respectively. In contrast, for bilateral fixation, the height decrease resulted in no significant change in ROM for flexion–extension (*P* = 0.686), lateral bending (*P* = 0.698), and axial rotation (*P* = 0.133). Using a short fusion cage, the load bearing decreased in 17.1, 21.5, and 54.1% (*P* < 0.05) for the cage alone, unilateral, and bilateral fixation, respectively.

**Conclusions:**

A cage longer than the intervertebral space should be chosen to increase the stability and intervertebral graft load borne when performing TLIF with unilateral instrumentation.

## Background

Unilateral pedicle screw–rod fixation is advocated as an alternative to bilateral fixation for lumbar fusion surgery [1, 2]. Minimizing the invasiveness and decreasing the construct stiffness are the two most important driving forces to modify lumbar instrumentation from the bilateral to the unilateral technique. It is reported that unilateral instrumentation is as effective as bilateral instrumentation for one- or two-level lumbar interbody fusion [1, 2]. However, some studies suggest the inferiority of unilateral fixation [3, 4].

The height of the interbody cage influences the segments stability and the fusion success. According to Bagby’s distraction–compression theory, an increasing distraction of the annulus fibrosus enhances intervertebral stabilization [5]. In addition, a biomechanical study shows that the degree of stabilization is related to the amount of disc space distraction [6]. From a clinical point of view, an insufficient cage height fails to restore the lumbar spine alignment and leads to cage migration and fusion failure [7]. Nevertheless, there is paucity of clinical and biomechanical evidence about the fusion cage height on the success of lumbar fusion.

Wolf’s law implies that bone fusion and remodelling is facilitated by a mechanical load borne by the bone. Moreover, it is established that load bearing is an osteogenic stimulus in animal models [8, 9]. Most of the previous biomechanical studies mainly focused on the stability of the unilateral technique. Thus, the load bearing of the fusion cage, which is an important influencing factor for successful fusion, has received little attention.

The purpose of this study is to quantify the role of cage height on the segmental flexibility and load sharing of the lumbar spine in the scenario of unilateral and bilateral instrumentation. Neither the superior nor inferior unilateral and bilateral fixation techniques were considered in our analyses. By comparing the flexibility and load sharing between the long and short cages configurations, we aim to provide a reference for fusion cage selection when performing lumbar interbody fusion with unilateral instrumentation.

## Methods

### Specimen preparation

Seven freshly-dissected and frozen human lumbar spine specimens (T12-S2) were used in this study. All the specimens were free from severe degeneration and spinal disease as assessed by gross visualization and radiographs. The initial intervertebral disc (IVD) heights were measured from the digital lateral X-ray images. The donors were four males and three females who aged 64.4 ± 9.2 years (median of 65 years, ages between 51 and 75 years) at the time of death (Table 1).

**Table 1:** Age, gender, initial IVD height, and tested cage height of the seven specimens

Specimen	Age (years)	Gender	Initial IVD height (mm)	Tested cage height	
				Long (mm)	Short (mm)
A	75	Male	13.2	14	12
B	65	Female	11.1	12	10
C	51	Male	11.7	12	10
D	72	Male	8.5	10	8
E	65	Female	9.7	10	8
F	53	Male	8.3	10	8
G	70	Female	9.2	10	8

The specimens were stored at −20 °C in double sealed plastic bags. Before testing, the specimens were thawed at 4 °C for 24 h. Most of the paraspinal muscle was removed from each specimen, whereas all the ligaments, joint capsules, intervertebral discs, and osseous structures were kept intact. A normal saline solution was intermittently sprayed on the specimens to keep them moist during the tests.

### Implants and instrumental configuration

To guarantee that the cages height was the only influencing factor on the biomechanical performance of the fused segments, two sets of box-shaped modular cages were respectively designed for the flexibility and load sharing tests.

**Fig. 1:**
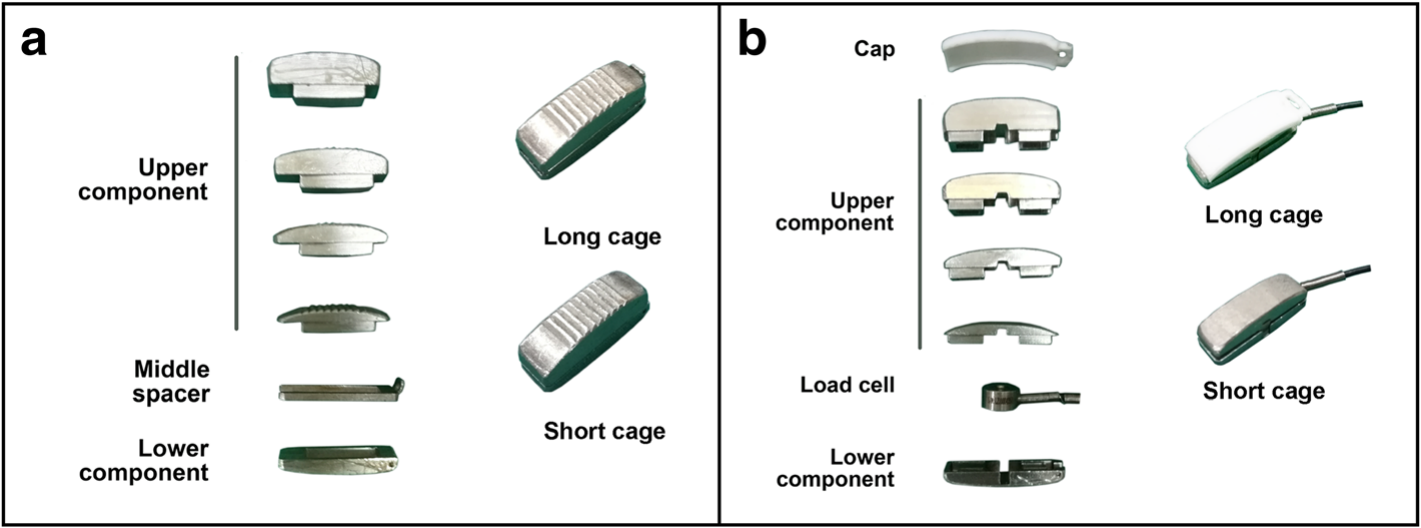
The customized cages used for flexibility (**a**) and load sharing (**b**) test

The cages used for the flexibility test were composed of three parts, namely, the upper component, the middle spacer, and the lower component. The middle spacer was 2-mm thick and simulated a short cage when removed (Fig. 1a).

The cages used for the load sharing test were composed of four parts, namely, the cap, the upper component, the load cell, and the lower component. The polyethylene cap was 2-mm thick and simulated a short cage when removed. A sub-miniature compression load cell (model FC10, Forsentek Co. Ltd., Shenzhen, China) was embedded between the upper and lower parts of the cage to record the load supported by the cage (Fig. 1b).

The long cage was set to have a higher height than the target IVD space, whereas the short cage had a lower height than the IVD space (Table 1).

The upper and lower surfaces of the custom cages were cambered to fit in the shape of intervertebral disc space. Both sets of cages were 10 mm in wide and 30 mm in length. The heights were 10/8 mm, 12/10 mm, 14/12 mm, 16/14 mm (with/withour middle spacer or cap) respectively (Fig. 1).

**Fig. 2:**
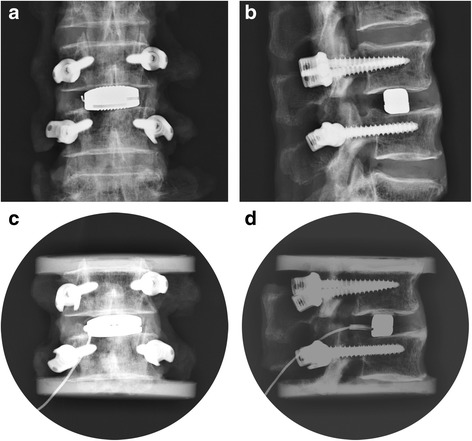
Cage position examined by X-ray. A long cage for flexibility test: AP view (**a**) and lateral view (**b**); A long cage for load sharing test, AP view (**c**) and lateral view (**d**)

A right-side transforaminal lumbar interbody fusion (TLIF) was carried out with a facetectomy at the L3/4 interval. The intervertebral space was prepared and the long cage was obliquely inserted and then adjusted to a transverse position by hammering on its tail (Fig. 2). Multiaxial pedicle screws of 6 mm (SINO fixation system, Weigao Co., China) were bilaterally implanted into the involved vertebrae, but the left-side screws were connected with rods only for bilateral fixation.

The configuration of long or short cage with unilateral, bilateral, or no posterior fixation were combined to stabilize the operated segment. A total of seven configurations were tested in this study:

1. An intact spine;
2. Long cage alone;
3. Long cage with unilateral pedicle screw–rod fixation;
4. Long cage with bilateral pedicle screw–rod fixation;
5. Short cage alone;
6. Short cage with unilateral pedicle screw–rod fixation;
7. Short cage with bilateral pedicle screw–rod fixation.

### Flexibility test protocol

**Fig. 3:**
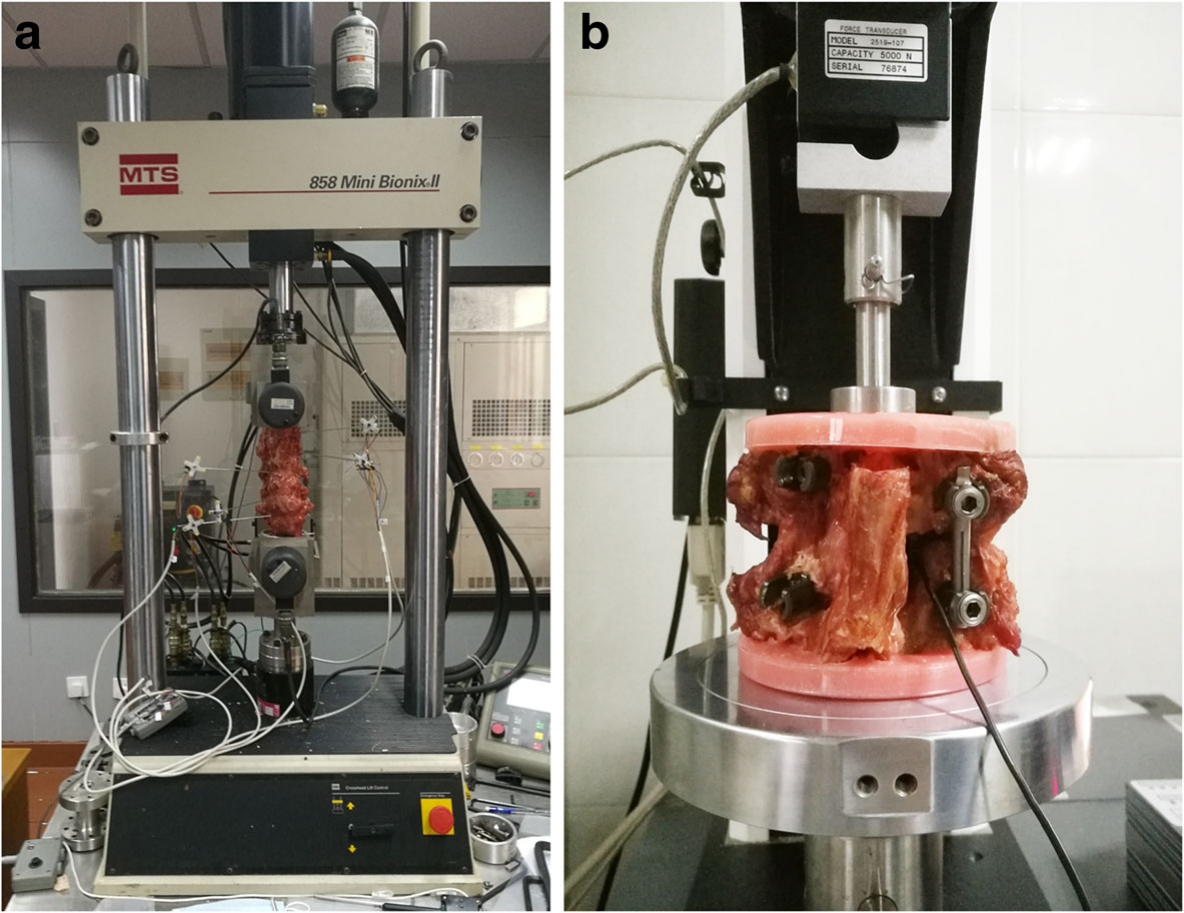
Picture of the flexibility (**a**) and load sharing (**b**) test setup. Note the cable for the load cell protruding from the intervertebral space in the load sharing test

For the flexibility test, the range of motion (ROM) was evaluated for flexion–extension, lateral bending, and axial rotation. The T12 and S1–2 vertebral bodies were embedded in customized flanges using acrylic resins, ensuring that the middle disc was horizontally aligned. Before the embedding process, four wood screws were placed into the upper and lower vertebrae in order to improve the fixation between the vertebrae and acrylic resins. The specimen was then fixed to the Spine Kinematics Sub-System (Model 608.33. MTS Systems Co., MN, USA) (Fig. 3a). This system acts in all the 6 DOF and a load cell was mounted on the upper gimbals for moment controlling. Hydraulic actuation enables precise control in the 4 active DOF to apply lateral bending and axial rotation. For the flexion–extension test, the specimen was rotated around the vertical axis in 90°.

The non-destructive flexibility was then tested with 50 N axial compressive loads and 7.5 Nm pure moments at a rate of 1 °/s in a sinusoidal pattern. A total of 10 moment cycles were applied with the first 7 to pre-condition the specimen and the last 3 to collect ROM data.

The motion of the vertebral bodies was tracked with the Optotrak Certus 3D measurement system (NDI International, Waterloo, Canada). Labelled markers using infrared light-emitting diodes were fixed to L1-L5 vertebral bodies with Steinmann pins. The dynamic rigid-body motion of the markers, which represented the labelled vertebral bodies, with respect to a global axis system was tracked. The NDI First Principles software was used for real-time collection and management of the measured data. The ROM denoted the flexibility of the segments, and was defined as the maximum rotational displacement (expressed in degrees) between the L3 and L4 vertebrae, following the direction of the applied moment. The stability was inversely proportional to the flexibility of the operated segment.

Given the inherent variability among specimens, the same specimen was used for comparison among different configurations. For each specimen, the seven configurations were tested in the order presented above, starting from the intact spine configuration, followed by the three long cage and the three short cage configurations.

### Load sharing test protocol

To verify load sharing, the L3–4 vertebrae from the specimens were moulded and mounted in a material testing machine (model 3345, Instron Co., MA, USA). Then, non-destructive uniaxial compression tests were conducted (Fig. 3b).

Axial compressive loads of 400 N were applied to the upper vertebra to simulate the load on L3–4 lumbar spine in the neutral, upright posture [10]. The ramp compression load increased until the target load was reached, and the load bearing of the cage was measured by the embedded load cell. The specimens were loaded for five times and data were collected from the last loading. The load data were read on a display controller (model FXT, Forsentek Co. Ltd., Shenzhen, China). The percentage of load bearing of the cage for each configuration was obtained from dividing the force measured at the cage by the compression force applied, and multiplying by 100%.

### Statistical analysis

The data of this study were processed using the SPSS 22.0 statistical software (SPSS Inc., Chicago, IL, USA). Descriptive data were presented as mean ± standard deviation. To validate the test protocol, repeated-measures one-way ANOVA was performed to compare the effect of posterior instrumentation on the flexibility and load sharing of the operated segments (four levels: intact spine, cage alone, UPS and BPS). When Mauchly’s test of sphericity was violated (*p* < 0.05), the Greenhouse–Geisser correction results were reported. Post hoc multiple comparisons were conducted with LSD’s test. To determine the effect of fusion cage height decrease on the biomechanical performance of the tested configurations, the paired t test was used for comparison between the long cage and short cage configurations. *P* < 0.05 values were considered as statistically significant.

The percentage of ROM increase was defined by 100% × (short cage configuration ROM - long cage configuration ROM)/ long cage configuration ROM.

## Results

### Protocol validation

**Fig. 4:**
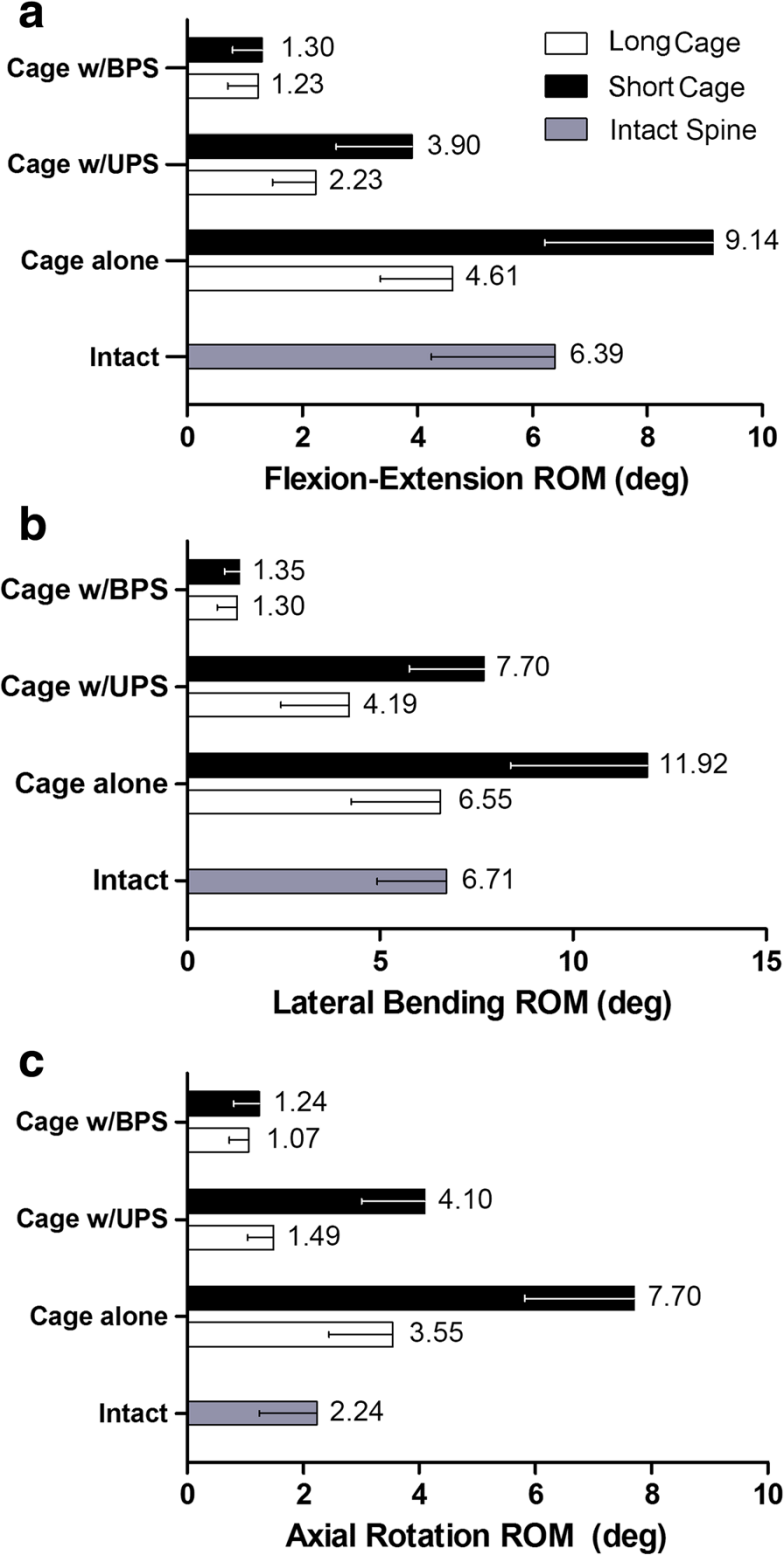
L3/4 segment ROM of the tested configurations

**Fig. 5:**
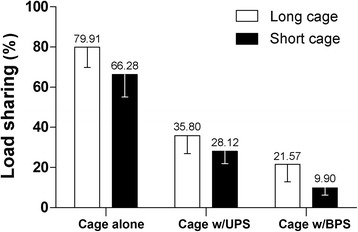
The load sharing of the tested configurations

For the flexibility test, using a long cage, the unilateral fixation allowed a wider ROM than the bilateral fixation (*P* < 0.05), but a lower ROM than the cage alone configuration (*P* < 0.05) (Table 2 and Fig. 4). For the load sharing test, a long cage received 79.91%, 35.80% and 21.57% of the total load in the cage alone, unilateral and bilateral fixation configuration, respectively. The differences were statistically significant for pairwise comparison (*P* < 0.05) (Table 2 and Fig. 5).

**Table 2:** Results of flexibility and load sharing tests for the treated lumbar specimens

		Long cage	Short cage	*P*-value*
Flexion and extension ROM (deg)^a^	Intact spine^b^	6.39(2.15)		
	Cage alone	4.61(1.25)	9.14(2.92)	0.002
	UPS	2.23(0.75)	3.90(1.31)	0.004
	BPS	1.23(0.53)	1.30(0.51)	0.686
Lateral bending ROM (deg)^a^	Intact spine^b^	6.71(1.79)		
	Cage alone	6.55(2.30)	11.92(3.53)	<0.001
	UPS	4.19(1.76)	7.7(1.95)	0.005
	BPS	1.30(0.52)	1.35(0.40)	0.698
Axial rotation ROM (deg)^a^	Intact spine^b^	2.24(1.00)		
	Cage alone	3.55(1.10)	7.7(1.88)	<0.001
	UPS	1.49(0.44)	4.1(1.09)	<0.001
	BPS	1.07(0.34)	1.24(0.44)	0.133
Load sharing (%)^a^	Intact spine^b^	–		
	Cage alone	79.91(10.03)	66.28(11.28)	0.022
	UPS	35.80(8.94)	28.12(6.20)	0.020
	BPS	21.57(8.70)	9.90(3.61)	0.022

*UPS* unilateral pedicle screw fixation, *BPS* bilateral pedicle screw fixation

*P value: Paired t test between Long and Short cage

^a^The data are given as mean, with standard deviation in parentheses

^b^The intact spine specimens were tested without a fusion cage implanted

### ROM

When the cage height decreased 2 mm, the segment flexibility with unilateral fixation showed a significant increase in the ROM for flexion–extension, lateral bending, and axial rotation of 74.9%, 83.8%, and 175.2%, respectively (*P* < 0.01) (Table 2 and Fig. 4). On the other hand, for bilateral fixation, the decreased cage height resulted in no significant changes in the ROM in flexion–extension (*P* = 0.686), lateral bending (*P* = 0.698), and axial rotation (*P* = 0.133) (Table 2 and Fig. 4).

### Load sharing

When the cage height decreased 2 mm, it showed a 21.5% (*P* < 0.05) and 54.1% (*P* < 0.05) decrease in load bearing for the unilateral and bilateral fixations, respectively. In the cage alone configuration, the short cage showed a decrease of 17.1% in load bearing compared with the long cage (*P* < 0.05) (Table 2 and Fig. 5).

## Discussion

There is an evolutive history of more than a hundred years on the treatment of spinal illness with spinal fusion surgery [11]. Colward introduced the posterior lumbar interbody fusion procedure in 1953 and Steffee supplemented the interbody fusion with pedicle screw and plate fixation in 1986. After that, interbody fusion with bilateral instrumentation was gradually accepted as the standard procedure for lumbar fusion surgery. With rigid instrumentation added to improve intervertebral stabilization, the complication of the procedure decreased and the fusion rate improved. In the recent years, unilateral pedicle screw fixation has been advised as an alternative to bilateral fixation for lumbar fusion [1, 2, 12]. Unilateral fixation not only reduced surgery trauma, but also provided similar results to bilateral fixation regarding fusion rates, total complications, and patient-reported functional outcomes. Nevertheless, given the inherent asymmetry and decreased strength of this system, using unilateral instrumentation may result in non-union, metal failure, or cage migration [3, 4, 13].

Firstly, we validated the flexibility test protocol. Using a long cage, unilateral instrumentation provided significantly lower flexibility than the cage alone configuration for the three tested motions, but offers higher flexibility than bilateral instrumented TLIF. These results were similar to previous researches on the biomechanics of unilateral instrumentation and validated the flexibility test protocol [14, 15]. Next, we tested the response of different posterior instrumentation to cage height decrease. With a short cage in place, unilateral instrumentation showed an increase of flexibility in all of the three tested motions. However, for the segment stabilized with bilateral fixation and for each test motion, no significant difference was found in the flexibility between the long and short cage configurations. These results indicated that insufficient height of the fusion cage will decrease the stability of the operated segments with unilateral fixation, but does not bring similar effect to the bilateral fixation configuration.

It was commonly held that, although lower than bilateral fixation, the stability provided by unilateral fixation is enough for fusion. Considering the sensitivity of unilateral fixation to cage height variation, the relatively low stability of unilateral fixation construct will be further decreased by a height insufficient fusion cage, so that the stability required for fusion could not achieve. Therefore, we believe that insufficient cage height might be one of the reasons for the failure of unilateral fixed interbody fusion. This suggests that a cage longer than the intervertebral space should be chosen for unilateral fixation.

A possible reason for the different response to cage height decrease between these two kinds of fixation is that the strength of unilateral fixation is relatively weak, and the distraction-compression mechanism resulted from cage insertion contributes substantially to the stabilization of the operated segments. However, the robust bilateral screw–rod fixation provided sufficient stiffness to stabilize the segments, and the cage made a relatively small contribution to the segments stability.

As this is the first study to investigate the load sharing of unilateral fixation, it was difficult to find similar study to validate the load sharing test protocol. This study shown a long cage received 79.91%, 35.80% and 21.57% of the total load in the cage alone, unilateral and bilateral fixation configuration, respectively. In theory, the stronger the posterior instrumentation is, the less load anterior fusion cage would bear. This result was conforming to prediction and could be used to validate the test protocol.

Increasing load borne by the intervertebral graft potentially promotes fusion success [8, 9]. In this study, we showed that the load borne of a long cage were significantly higher than the load borne of a short cage in both unilateral and bilateral fixation configurations. This indicated a long cage would reduce the stress shielding of posterior instrumentation. This increased anterior column loading could be a fusion promoting factor, because of the compression-related bone-healing enhancing that is generated during the assumption of the upright posture.

Interestingly, we found the annulus fibrosus could shield a short cage from load bearing. With a short cage in place, we found that the cage load in both the unilateral and bilateral fixations decreased significantly compared with the corresponding long cage configurations. It is clear that the posterior metal support showed stress shield to the anterior cage [16]. However, in the cage alone configuration, without any metal support shield, there was still a significant decrease of the cage load when the cage height decreased. Because in the cage alone configuration, without screw-rod support, the annulus fibrosus was the only structure, except from the fusion cage, to support the perpendicular compressive load in the IVD, we postulate that the annulus fibrosus shielded the short cage from load bearing. In order to facilitate interbody graft load sharing, it is recommended that a cage longer than the target IVD space should be chosen when performing interbody fusion surgery.

This study has several limitations. Firstly, the contribution of paravertebral muscles and creep deformation of the specimen to the biomechanical performance of the operated segments was not taken into account. Therefore, the in vitro biomechanical test might be too simple to perfectly replicate the complicated in vivo kinematics and load sharing characteristics of lumbar spine. Secondly, the sample size was relatively small and limited to an aged population, there may be senile bone mass loss that would make this population different from other populations. Lastly, while it is suggestive that increasing initial stability of the fusion segment and load bearing of the fusion cage promote a success fusion, there is a paucity of direct clinical evidence to prove this relationship. It is needed that further clinical investigations on the effect of cage height on clinical efficacy of unilateral fixation construct.

## Conclusion

An insufficient height of the cage significantly decreases the stability of the operated segments with unilateral fixation but does not bring significant influence to the bilateral fixation configuration. The load borne of a long cage is significantly higher than the load borne of a short cage. Thus, it is suggested that a cage longer than the intervertebral space should be chosen to increase the stability and intervertebral graft load borne when preforming TLIF with unilateral instrumentation.

## Abbreviations

Cage w/BPS: Cage with bilateral pedicle screw fixation
Cage w/UPS: Cage with unilateral pedicle screw fixation
DOF: Degrees of freedom
IVD: Intervertebral disc
ROM: Range of motion
TLIF: Transforaminal lumbar interbody fusion
